# Emotion regulation, attention to emotion, and the ventral attentional network

**DOI:** 10.3389/fnhum.2013.00746

**Published:** 2013-11-07

**Authors:** Roberto Viviani

**Affiliations:** ^1^Department of Psychiatry and Psychotherapy III, University of UlmUlm, Germany; ^2^Institute of Psychology, University of InnsbruckInnsbruck, Austria

**Keywords:** emotion regulation, attention to emotion, ventral attentional network, thought control, scrambled sentences test, dual-process models

## Abstract

Accounts of the effect of emotional information on behavioral response and current models of emotion regulation are based on two opposed but interacting processes: automatic bottom-up processes (triggered by emotionally arousing stimuli) and top-down control processes (mapped to prefrontal cortical areas). Data on the existence of a third attentional network operating without recourse to limited-capacity processes but influencing response raise the issue of how it is integrated in emotion regulation. We summarize here data from attention to emotion, voluntary emotion regulation, and on the origin of biases against negative content suggesting that the ventral network is modulated by exposure to emotional stimuli when the task does not constrain the handling of emotional content. In the parietal lobes, preferential activation of ventral areas associated with “bottom-up” attention by ventral network theorists is strongest in studies of cognitive reappraisal. In conditions when no explicit instruction is given to change one's response to emotional stimuli, control of emotionally arousing stimuli is observed without concomitant activation of the dorsal attentional network, replaced by a shift of activation toward ventral areas. In contrast, in studies where emotional stimuli are placed in the role of distracter, the observed deactivation of these ventral semantic association areas is consistent with the existence of proactive control on the role emotional representations are allowed to take in generating response. It is here argued that attentional orienting mechanisms located in the ventral network constitute an intermediate kind of process, with features only partially in common with effortful and automatic processes, which plays an important role in handling emotion by conveying the influence of semantic networks, with which the ventral network is co-localized. Current neuroimaging work in emotion regulation has neglected this system by focusing on a bottom-up/top-down dichotomy of attentional control.

## Introduction

Emotion and emotion regulation are important issues in clinical neurosciences because disturbed affect, impulsivity, and low control capacity are common in psychopathology. Evidence gathered from diverse strands of research, behavioral as well as based on neuroimaging methods, has shown attention to be a key mechanism for the achievement of regulatory goals. A first type of studies has shown that the manipulation of attentional load by varying cognitive processing demands may alter responses to emotional stimuli (Hariri et al., 2000; Pessoa et al., 2002; Compton et al., 2003; Banich et al., 2009; Luo et al., 2010). These studies demonstrated that increasing attentional demands (for example by varying the difficulty of the task) may attenuate response to emotional stimuli, even if the task does not require diverting attention from the emotional aspect of the stimulus set. A second strand of research has used neuroimaging to investigate the neural correlates of asking individuals to attend to their own internal emotional state and modify it in a specific direction (Schaefer et al., 2002; Lévesque et al., 2003; Ochsner and Gross, 2005; Beauregard, 2007; Wager et al., 2008). This form of explicit emotional control is often referred to as “voluntary” or “deliberate” emotion regulation (Gross and Thompson, 2007). Cognitive control mechanisms may also be important to keep thoughts out of mind that are concerned with emotional issues (Wenzlaff and Wegner, 2000; Brewin and Beaton, 2002). Additional evidence on the importance of attention for regulation has been provided by studies that considered the developmental link between emergence of attentional capacity and behavioral self-control (Diamond and Gilbert, 1989; Posner and Rothbart, 1998, 2000). Finally, a large number of behavioral studies have documented the effects of attending to stimuli with emotional tone (for reviews, see Mathews and MacLeod, 1994; Bradley, 2009; Yiend, 2010) and the existence of emotion-congruent attentional biases in psychopathology of affect (Williams et al., 1997; Yiend, 2010), thus underscoring the importance of the interaction between emotional and attentional processes in psychopathology.

**Figure 1:**
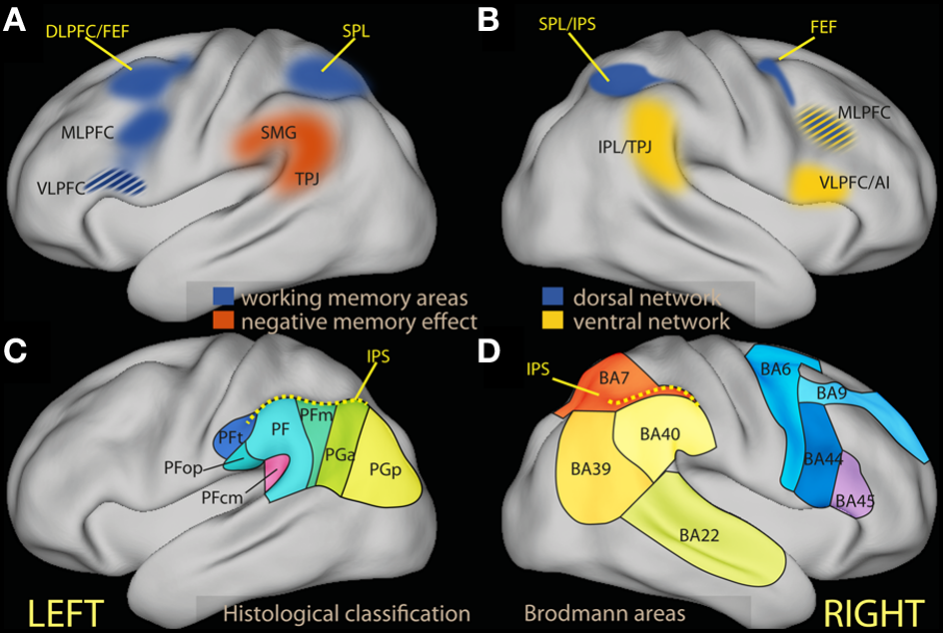
**(A)** Schematic diagram of regions associated in the left hemisphere with working memory tasks (in blue) and in negative subsequent memory effects [in red, drawn after review data presented by Uncapher and Wagner (2009)]. The ventrolateral region of the prefrontal cortex is in many studies associated with executive tasks, and its belonging to processes attributed here to the dorsal network is unclear (see text). **(B)** Schematic diagram of areas associated with the dorsal (in blue) and the ventral attentional network [in yellow; drawn after the review data of Corbetta and Shulman (2002) and Corbetta et al. (2008)]. **(C)** Schematic partition of the inferior parietal region based on cortical laminar organization in man [approximate drawing based on Caspers et al. (2006)]. **(D)** Schematic partition of the parietal and the relevant prefrontal lobes in Brodmann areas. AI, anterior insula; DLPFC, dorsolateral prefrontal cortex; FEF, frontal eye fields; IPL, inferior parietal lobule; IPS, inferior parietal sulcus; MLPFC, middle frontal gyrus; VLPFC, ventrolateral prefrontal cortex; SMG, supramarginal gyrus; SPL, superior parietal lobule; TPJ, temporoparietal junction.

The insight emerging from functional neuroimaging has been the recognition of the existence of distinct brain circuits that are activated on the one hand by the perceptual encoding of emotional stimuli, and on the other hand by regulatory processes. The amygdala (a brain structure part of the limbic system located in the medial anterior temporal lobe) has been consistently involved in the early perception of emotionally arousing stimuli (Davis and Whalen, 2001; Dolan, 2002; Dolan and Vuillemier, 2003; Vuilleumier, 2005; Adolphs and Spezio, 2006). Specific mechanisms have been shown to operate on this early perception to refocus attention effectively (Dolan, 2002; Dolan and Vuillemier, 2003; Vuilleumier, 2005). These mechanisms allow emotional material to undergo preferential processing (Vuilleumier, 2005; Phelps, 2006), prioritizing the handling of information that is likely to be relevant for the goals and the survival of the individual. In contrast, the control of emotional stimuli interfering with cognitive tasks is associated with activation of the prefrontal areas that are recruited during cognitive control and mnemonic encoding of general stimuli (Elliott et al., 2000; Compton et al., 2003; Banich et al., 2009). These areas have been shown by studies of cognition to be involved together with medial prefrontal and parietal areas in a dorsal cortical network critical to cognitive control (Rypma and D'Esposito, 1999; Smith and Jonides, 1999; Wager and Smith, 2003; Owen et al., 2005; Figure 1A), active when attending and giving priority to stimuli according to internal goals, rather than the perceptual or emotional salience. Consistently with their prominent role in cognitive control, these same prefrontal areas are also involved in studies of voluntary emotion regulation (see Ochsner and Gross, 2008 and Ochsner et al., 2012 for review).

Interestingly, theorists of emotion regulation also mention the existence of “automatic emotion regulation” forms (Mauss et al., 2007; Phillips et al., 2008) or of unconscious varieties of emotion regulation (Bargh and Williams, 2007), which contrast with the voluntary form just mentioned. The term automatic refers here to processes evoked by the stimulus and running without monitoring (Mauss et al., 2007; Gyurak et al., 2011), or initiated without awareness and not subject to strong capacity limitations (Williams et al., 2009). However, almost all existing studies have focused on forms of emotion regulation mediated by top-down regulatory processes. The resulting account of emotion regulation is based on a dual-process model (Barrett et al., 2004) that opposes automatic sensory encoding of emotionally arousing stimuli on the one hand, and on the other an integration of attentional mechanisms and prefrontal function envisaged to account more generally for cognitive control processes (Posner and Rothbart, 1998; Posner et al., 2002; Compton, 2003; Ochsner et al., 2009a; Hofmann et al., 2012).

This account is consistent within biased competition theories of attention (Desimone and Duncan, 1995; Miller and Cohen, 2001). Emotionally arousing stimuli in bottom-up perceptual channels may be viewed as particularly effective in competing for access to short-term memory (Vuilleumier, 2005; Stanley et al., 2009), requiring strong bias by top-down processes to maintain cognitive control. Accordingly, emotion dysregulation may be seen as arising from increased reactivity to emotional stimuli (mapped to increased activation of structures such as the amygdala) or from the failure to down-regulate emotional representations through the biasing activity of prefrontal areas involved in voluntary cognitive control (Posner and Rothbart, 1998; Phillips et al., 2003a,b; DeRubeis et al., 2008). In the following, we will refer to this dual-process model as to the “accepted view” of emotion regulation, since it is the one that informs most neuroimaging studies of emotion regulation and its failure in psychopathological conditions. Considerable evidence has now been gathered in neuroimaging studies on the involvement of the amygdala in the psychopathology of affect and impulsivity (Rauch et al., 2000; Herpertz et al., 2001; Whalen et al., 2002; Siegle et al., 2007), while the data on the involvement of the prefrontal areas in the psychopathology of affect are less univocal (Fitzgerald et al., 2006; Taylor and Liberzon, 2007; Thomas and Elliott, 2009).

In recent years, the dual-process model has been extensively investigated by neuroimagers. An important debate has involved the influence of top-down processes at early stages of sensory encoding of emotional stimuli, or their relative automaticity (Okon-Singer et al., 2007, 2012; Pessoa, 2008; Vuilleumier and Huang, 2009; Pessoa and Adolphs, 2010; Tamietto and de Gelder, 2010; Dolcos et al., 2011). These influences suggest that the form of automaticity of early sensory encoding in the amygdala and visual pathways consists of the low level of control minimally required for its processing. This demonstrates the importance of refining the simple dichotomy between automatic and controlled processes (Neumann, 1984), as in views emphasizing the gradualism of features defining automaticity (Moors and De Houver, 2006). Another important issue is the capacity of the amygdala and subcortical processing to prime visual cortex for processing emotionally salient stimuli, allowing the early perception of emotional stimuli to refocus attention effectively (Dolan, 2002; Dolan and Vuillemier, 2003; Vuilleumier, 2005; Bach et al., 2011). These refinements document the complex interaction between bottom-up sensory encoding and top-down control during appraisal and regulation of emotion, but do not question the dual-process character of the model.

A possibly greater challenge to dual-process models has emerged in neuroimaging studies of spatial attention. These studies originally set out to investigate the functional properties of areas known to be involved in hemispatial neglect, a syndrome affecting patients with parietal damage (Corbetta et al., 1993). In the last years, these studies have provided increasing evidence of the existence of a ventral network in many respects opposed, but also interacting with the dorsal attentional network in ensuring the correct functioning of attentional processes (Corbetta and Shulman, 2002; Corbetta et al., 2008; Shulman et al., 2010). Of course, it has been held for a long time that different forms of attention may be distinguished (James, 1890), and more specifically, that executive attention and orienting may be differentiated both functionally and on the basis of the associated brain circuits (Posner, 1980; Jonides, 1981; Müller and Rabbitt, 1989; Posner and Petersen, 1990). However, the ventral network model of attentional processes differs from previous accounts because of the description of a new class of stimuli capable to trigger orienting, and of the functional differentiation of ventral and dorsal areas (as in the parietal lobe), which act concurrently and in a coordinated fashion. Furthermore, the importance of the ventral network is emphasized even when deactivated during the execution of a focused task. As argued more extensively below (Section The Dorsal and the Ventral Attentional Networks: the Issues for Emotion Regulation), the evidence on the role of the ventral network gathered in studies of cognition suggests that at least three separate processes, instead of two, are involved in the interaction between incoming stimuli and internal goals in determining the focus of attention.

The description of a ventral attentional process, concomitantly engaged and interacting with attentional processes of executive nature, raises the question of the role played by the attentional orienting mediated by the ventral network in emotion processing and emotion regulation. This question does not by itself challenge the relevance of executive processes and their neural correlates for the deliberate control of emotion, as the evidence in this respect, briefly mentioned above, is quite extensive. Rather, it draws attention to the extent to which existing observations may carry evidence for an involvement of the ventral network that has so far eluded systematic analysis. Similarly, this question does not challenge the evidence on the effect of emotional material on the early sensory processing of stimuli. What this question does challenge, however, is the adequacy of the dual-process attention-based model to account fully for the data and the phenomenology of the interaction between emotion and attention and of emotion regulation.

In this review I will first summarize findings on the ventral attention network with the aim of highlighting the questions they raise for the dual-process view of emotion regulation and its neurobiological basis. These findings draw attention to the importance of understanding the characteristics of stimuli that activate the ventral attentional network, which belong to a category referred to as “behaviorally relevant.” I will then review studies of spatial attention and memory that considered the specific effect of emotional stimuli to see if they provide any evidence on their propensity to activate this network and fall within this category. In a second step, I will review studies on voluntary and spontaneous emotion regulation, showing that some of their findings are difficult to account for in the dual process view of emotion regulation, but are compatible with a model of attentional processes that includes the specific role of the ventral network. These studies suggest that the neural substrates of forms of emotion regulation that are part of a spontaneous process, or emerge in the absence of a tightly constrained task set, overlap with the ventral, but not the dorsal, network. These results motivate the thesis advanced here that the ventral attentional network implements a form of attentional control of high importance for affective functioning, and that the state of activity of the ventral network reflects the absence or existence of proactive control processes, corresponding to the absence or existence of a task set targeting the influence of emotion on response. I will also introduce recent evidence suggesting that, in the absence of proactive control, the ventral network may steer response through modalities that differ from those of voluntary emotion regulation, and that may operate when regulation takes place spontaneously, i.e., in the absence of explicit, voluntary efforts, or arises from the spontaneous elaboration of presented stimuli.

## The dorsal and the ventral attentional networks: the issues for emotion regulation

First described in the right hemisphere in studies of spatial attention, the ventral network interrupts and resets attention to behaviorally salient stimuli, while the task of the dorsal network is to maintain the locus of attention in the face of distraction, select stimuli according to prior information or goals, and coordinate responses (Corbetta and Shulman, 2002; Corbetta et al., 2008). The dorsal network includes dorsal frontal areas near or at the frontal eye fields (FEF), related to the dorsolateral prefrontal cortex (DLPFC), and the intraparietal sulcus (IPS) and the superior parietal lobule (SPL) in the parietal lobe (Figure 1B, blue). The DLPFC constitutes the neural substrate of top-down control processes associated with limited capacity (Posner and Presti, 1987), executive (Kane and Engle, 2002; Baddeley, 2003) and biased attention models of cognitive control (Desimone and Duncan, 1995; Miller and Cohen, 2001). The parietal node of the dorsal network is co-activated by recruitment of these top-down processes, but (at least in studies of spatial attention) is also active when attention is reoriented by external stimuli due to their sensory salience (Corbetta et al., 1993; de Fockert et al., 2004). Unlike the dorsal network, the ventral network is not activated by representations of goals or expectations, but responds together with the dorsal network when initially unattended objects of relevance to the task are detected (Corbetta et al., 2000). Core regions of the ventral network are the inferior parietal lobule (IPL) and the adjacent temporo-parietal junction (TPJ). In the prefrontal lobe, studies of spatial attention have associated the ventrolateral prefrontal cortex/inferior frontal gyrus (VLPFC/IFG) and the anterior insula with the ventral network (Corbetta et al., 2008; Figure 1B, yellow).

Unlike the dorsal network, activity in the ventral network is reduced during focussed tasks relative to fixation or baseline. However, the ventral network can be briefly activated together with the dorsal network in circumstances in which attention is refocused by external stimuli of behavioral relevance (Corbetta et al., 2008). This observation gave rise to the hypothesis that deactivation has a functional interpretation as a form of filter on the perceptual and semantic encoding of the stimuli (Todd et al., 2005; Shulman et al., 2007). Because activation in the dorsal network is implicated in maintaining expectations of incoming stimuli, the deactivation may originate in the interaction with the dorsal network (Corbetta et al., 2008). Below, I refer to such task set-related processes as “proactive control.”

An early hypothesis formulated by spatial attention researchers is the applicability of their findings, in suitably generalized form, to selection and attendance of thoughts and memories in a semantic space (Posner, 1980). The inferior parietal cortex, in particular, hosts multimodal semantic association areas (Downar et al., 2000), and appears to be involved in perceptual tasks of apparently diverse nature (Husain and Nachev, 2007). Some of these tasks, such as oddball or go/no-go paradigms, involve the detection of salient items within a longer sequence (Linden et al., 1999; Clark et al., 2000; Marois et al., 2000; Downar et al., 2002). This variety of sources of parietal activation is consistent with findings in non-human primates. Recordings of firing activity in parietal neurons have provided evidence of a role of parietal cortex in dynamically computing priorities guiding selection not only for spatial orienting, but also for abstract rule-based actions (Gottlieb, 2006; Andersen and Cui, 2009; Bisley and Goldberg, 2010; Freedman and Assad, 2011). These recent studies confirm the topicality of traditional views of parietal cortex as a sensory integration or multimodal association area (Critchley, 1953) hosting abstract representations of external space (Bisiach et al., 1979; Mesulam, 1981). They show that the common denominator of parietal involvement is the integration of visuospatial and behavioral information from different sources, irrespective of whether response involves eye or limb movements or goal-directed choice. These data support the extension of models developed in studies of spatial attention to more general forms of computation of priorities for the generation of responses.

In man, a dorsal/ventral parietal dissociation in the left parietal region with several features analogous to those present on the right has been identified when selecting internal representations arising during memory tasks (Cabeza et al., 2008, 2012; Ciaramelli et al., 2008; Uncapher and Wagner, 2009; see, however, Sestieri et al., 2010) and when detecting changes in stimuli in the absence of a specific task (Downar et al., 2000). In neuroimaging studies of episodic memory, activation in the IPL at memory encoding has been noted to correlate with lower later recall (Figure 1A, red; for a systematic review, see Uncapher and Wagner, 2009). These negative memory effects (negative correlation between memory performance and IPL activity) are thought to result from slip-ups of processing away from the information to be encoded (Otten and Rugg, 2001; Wagner and Davachi, 2001). Led by the hypothesis that the IPL may be associated with shifts of spatial attention away from the focus (Corbetta and Shulman, 2002), memory researchers have proposed an analogous role for IPL in memory (Cabeza et al., 2008; Ciaramelli et al., 2010), leading to processing task-irrelevant thoughts or stimulus features (see also Li et al., 2007 and Congdon et al., 2010 for analogous results in attentional tasks). In these accounts, the attentional function of the ventral network is clearly different from the maintenance of goal processing in the face of distraction or interference attributed to executive attention. Rather, IPL appears here to be associated with internal reorienting as kind of attentional interrupt, leading to disengagement from the current focus and turning attention to a new item. In some cases, this reorienting function may contribute to performance by favoring processing atypical or infrequent aspects of the stimulus (Uncapher and Wagner, 2009), which may be of advantage in interaction with goal-oriented processing in a complex and varying environment. In other cases, this reorienting function may lead to performance decrements. In this body of literature, it is customary to refer to “top-down” and “bottom-up” attention to refer to the goal-directed allocation of attention to anticipated memory targets and to the orienting attention to automatically recollected memories (Cabeza et al., 2012). Here, ventral network activity is associated with processing an internally generated distracter.

However, researchers of spatial attention have also pointed out the capacity of the ventral network to steer attention irrespective of the perceptual salience of the stimulus or distracters. Here, the ventral network appears to control attention in the face of sensory salience. In dual-process accounts, top-down control fails when the high salience of a stimulus leads to winning the competition even in the face of top-down bias (Yantis and Jonides, 1984; de Fockert et al., 2004). In ventral network orienting, in contrast, it is rather the relevance to behavioral goals or long-term memory associations that determines refocusing of attention (Downar et al., 2001; Serences et al., 2005; Kelley and Yantis, 2010; Cabeza et al., 2012). This shows that the kind of salience that activates the ventral network differs from sensory salience, and appears to reflect both broad representations of active goals and long-term information. Indeed, ventral network orienting has been shown in specific conditions to override orienting to sensory salience (Indovina and Macaluso, 2007). Recent data show that reorienting can also take place to stimuli that have been made previously relevant by reward conditioning (Anderson et al., 2011; for a review of related findings, see Awh et al., 2012). This suggests that the salience encoded in ventral network areas may not only be defined online by the current task, but also by emotional experience.

Because ventral network-based refocusing of attention dissociates functionally and anatomically from both sensory salience-based orienting and executive attention (Corbetta et al., 2008; Anderson et al., 2011; Cabeza et al., 2012), a comprehensive model of attention that includes ventral network orienting must differ from a dual-process theory of control as exemplified by the accepted view of emotion regulation. In the following sections I intend to address two issues ensuing from the dissociation of dorsal and ventral networks. The first is the evidence that emotional content modulates activation in areas of the ventral network. A positive answer would suggest that emotional tone belongs to the category of “behavioral relevance” attributed to stimuli that capture attention through the ventral network. To see if emotional material tends to recruit the ventral network more than neutral material, I will review studies on the effect of emotional material on attention and memory in Section Neuroimaging Studies of Attention to Emotion and Memory below. The second issue is what kind of control function the ventral network may implement when applied to emotional content. This issue will be addressed by reviews on the differential involvement of dorsal and ventral areas in studies of emotion regulation in sections Neuroimaging Studies of Voluntary Emotion Regulation and Thought Control and Spontaneous Avoidance of Negative Material.

Although the ventral network described in studies of spatial attention extends to ventral right prefrontal areas, the present review will be restricted to the parietal region. One reason for this focus is that the involvement of this hub of the ventral network is well-documented in several tasks. In particular, a parallel dissociation between dorsal and ventral areas has been characterized not only in the right hemisphere in spatial attention studies, but also in the left in studies of memory (Cabeza et al., 2012). Another reason is the complexity of the debate on the function of ventral prefrontal regions in the left hemisphere. While some have provided evidence for the involvement of ventral prefrontal areas in short-term memory tasks, in contrast to involvement of dorsal regions in more demanding working-memory tasks (Rypma and D'Esposito, 1999; Rypma et al., 1999), others have shown its involvement in executive tasks with high degree of interference between stimuli (Demb et al., 1995; Thompson-Schill et al., 1997; Wagner et al., 1997; Jonides et al., 1998; for a recent overview, see Schulz et al., 2009). In the right hemisphere, VLPFC/IFG is active in tasks where response needs be inhibited (for reviews, see Aron et al., 2004; Chambers et al., 2009), but the specificity of this observation is called into question (Sharp et al., 2010). These are complex issues that require more attention than can be given here.

The inferior parietal region is a relatively large area that in man may be differentiated further on the basis of the laminar organization of the cortex (Figures 1C,D; Caspers et al., 2006, 2013), corresponding to different preferential connectivity patterns (Caspers et al., 2011; Mars et al., 2011). As this differentiation suggests, there are functional specializations within this region (Hutchinson et al., 2012; Mars et al., 2011). However, the focus of the present review is on the general characterization of the cognitive processes that may be hosted here, in contrast with those associated with dorsal regions. Drawing on views from neuropsychology (Mesulam, 1981) and the non-human primate literature (Gottlieb, 2006; Andersen and Cui, 2009; Bisley and Goldberg, 2010; Freedman and Assad, 2011), I will view the parietal region as involved in computing priorities for attentional selection and choice from information from different modalities, based on both sensory salience and behavioral relevance. This view is not inconsistent with the existence of subregions that are further specialized, for example by the type of information they integrate. The notion of ventral attentional network draws on neuroimaging data in man that suggest sensory salience and top-down control to map onto the dorsal, behavioral relevance onto the ventral portion of the parietal lobule.

## Neuroimaging studies of attention to emotion and memory

The studies of attention to emotion that will be considered here are those where emotional stimuli are used as cues (as in spatial attention paradigms) or as distracters, or are present in the stimulus set without providing information for the task. This restriction is justified by the consideration that, if emotional content is selected on the basis of a voluntary effort, there is no reason to assume that this selection cannot take place on the basis of processes instantiated in the dorsal attentional network (i.e., the dual-process and the alternative model presented here lead to the same prediction). Studies will be considered that report on the contrast between the effect of emotional and non-emotional stimuli. This contrast provides evidence on a specific effect of emotional material on the ventral network, not just on the possible activation of the ventral network during the task.

### Spatial attention tasks

Relatively few neuroimaging studies in the healthy have combined spatial attention paradigms with emotional stimuli (Table 1). A few studies used emotional stimuli as non-informative cues presented simultaneously to elicit covert reflexive orienting to the emotionally salient cue. The main reasoning behind these studies is contrasting emotional and neutral non-informative cues to investigate covert orienting to emotional cues as a manifestation of preferential processing. Two studies using emotional stimuli conditioned to aversive events found activations that extended from the IPS into the supramarginal gyrus and anteriorly toward the secondary sensory cortex (Fredrikson et al., 1995; Armony and Dolan, 2002). In contrast, a recent study in which the cue was conveying information about the magnitude of reward reported only weak or no effect on ventral parietal or temporoparietal regions (Tosoni et al., 2013). In this study, however, the cue could not be ignored, as it instructed on the position of the target. Pourtois et al. (2006) is a neuroimaging study of particular relevance to the present issue, since it looked at effects of non-informative cues explicitly to disentangle effects in the dorsal and ventral attentional systems using fearful and happy faces. They found that fearful emotional cues additionally activated a temporo-parietal-occipital region, which was associated with changes of activation in the dorsal attentional system at the presentation of the target. The locations in the temporo-parietal region were more posterior than those of the previous two studies. However, the emotional salience of the Pourtois et al. (2006) study was not acquired through conditioning, but presumably biologically determined.

**Table 1:** **IPL involvement in studies of attention to emotion**.

**Study**	**Task**	**Material**	**Lat.**	**IPL effect**
**SPATIAL ATTENTION TASKS**				
Fredrikson et al., 1995	Comparison of conditioned aversive stimulus after conditioning vs. without conditioning	Pictures	L~R	Yes
Armony and Dolan, 2002	Dot probe covert attention task with two non-informative cues, one aversively conditioned the other not	Cue	L~R	Yes
Pourtois et al., 2006	Dot probe with two non-informative cues, one intrinsically emotional (facial expression) one not	Cue	L<R	Yes (cue only trials)
Tosoni et al., 2013	Dot probe with cues additionally conveying information about expected reward	Cue		No
**INTERFERENCE TASKS**				
Vuilleumier et al., 2001	Flanker task with neutral images or faces alternating in the target/flanker position	Pictures		No
Ochsner et al., 2009b	Flanker task with emotional distractors	Words	L	No (compatible with deactivation)
Kanske and Kotz, 2011a	Simon task with emotional verbal (auditory) distractors	Words	R	No
Luo et al., 2007	Distracters in rapid serial visual presentation (RSVP)	Words	L>R	Yes +
Mitchell et al., 2008	Distracters in rapid serial visual presentation (RSVP)	Pictures	L~R	Yes +
Dolcos and McCarthy, 2006	Distracters during the delay of a short-term memory task	Pictures	L<R^*^	No (deactivation)
Erk et al., 2007	Distracters during the delay of a short-term memory task	Pictures		No
Dolcos et al., 2008	Distracters during the delay of a short-term memory task	Pictures	L~R	No (deactivation)
Chuah et al., 2010	Distracters during the delay of a short-term memory task	Pictures		No (deactivation)
Denkova et al., 2010	Distracters during the delay of a short-term memory task	Pictures	R	No (deactivation)
Oei et al., 2011	Distracters during the delay of a short-term memory task	Pictures	L~R	No (deactivation)
Iordan et al., 2013	Distracters during the delay of a short-term memory task	Pictures	L<R^*^	No (deactivation)
Yamasaki et al., 2002	Distracters between stimuli of a Go/No go task	Pictures		No
Whalen et al., 1998a	Emotional Stroop	Words		No
Compton et al., 2003	Emotional Stroop	Words	L>R	Yes +, negative valence only
Malhi et al., 2005	Emotional Stroop	Words	NA	Yes
Mitterschiffthaler et al., 2008	Emotional Stroop	Words		No
Wingenfeld et al., 2009	Emotional Stroop	Words		No
Shafer et al., 2012	Emotional Stroop	Words		No
**COGNITIVE/MOTOR INHIBITION**				
Elliott et al., 2000	Go/No go with emotional content as criterion	Words		No
Schulz et al., 2009	Go/No go with emotional content as criterion	Pictures		No
Goldstein et al., 2007	Emotional features (negative or positive) in standard Go/No go	Words	L>R	Yes +
Brown et al., 2012	Emotional features (negative or neutral) in standard Go/No go	Pictures	L<R	Yes +
Krebs et al., 2011	Emotional features (reward conditioning) in standard Stroop	Words	L>R	Yes
Haas et al., 2006	Congruent/incongruent faces + words	Both	L	No
Egner et al., 2008	Congruent/incongruent faces + words	Both	L~R	No (weak deactivation)
Lim et al., 2008	Congruent/incongruent faces + written data	Both	L	Yes
Park et al., 2008	Congruent/incongruent faces + words	Both	L	No (deactivation)
Chechko et al., 2009	Congruent/incongruent faces + words	Both	L	Yes

Lat, laterality of effect; R, right; L, left; L~R, about equal laterization; L<R, predominantly right-lateralized; L>R, predominantly left-lateralized; the asterisk marks lateralizations relative to deactivations. IPL finding, report of an effect in the inferior parietal lobe or in the temporo-parietal region. The symbol “Yes +” indicates that the IPL effect was the strongest across the brain. Effects reported for the comparison emotion vs. neutral (either directly, or in an interaction/simple effects contrast, as appropriate). For criteria of selection of studies in the table, see the Methods section.

**Figure 2:**
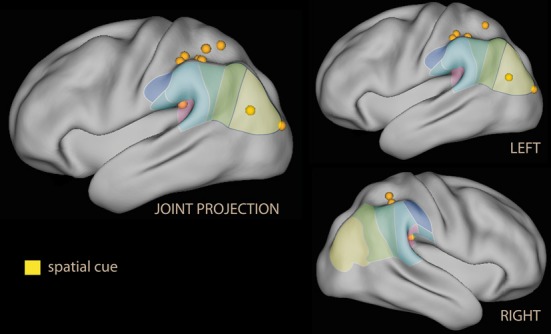
**Lateral rendering of foci for spatial attention studies with spatial cues with emotional tone (in yellow) on the PALS atlas (Van Essen et al., 2001)**. On the surface of the rendering, histological classification of the inferior parietal lobe in man (from Caspers et al., 2006; cf. Figure 1C). On the left, joint projection of foci from both hemispheres; on the right, separate rendering for left and right hemispheres.

In summary, these findings do suggest that emotional material in non-informative cues co-activates the ventral attentional system albeit in different parts of the IPL depending on the nature of the emotional salience. As in the spatial attention studies, activation of the ventral and dorsal systems occur together (both the ventral and the dorsal system were activated in parietal areas, Figure 2).

### Distracter tasks

A larger number of studies have investigated the effect of attention to emotion in tasks originally devised to study cognitive interference. In these studies, emotional stimuli appear as distracters. In biased competition models of attention, these stimuli require more top-down effort to be suppressed. Here, however, the focus will be on their capacity to co-recruit the IPL, the parietal hub of the ventral network associated with attentional capture on the basis of the “behavioral relevance” of the stimulus. In some studies, distracters are physically distinct, but spatially or temporally contiguous to the target stimuli; in others, the emotional tone is present as a dimension of the stimulus that is of no relevance for the formulation of the task response.

**Figure 3:**
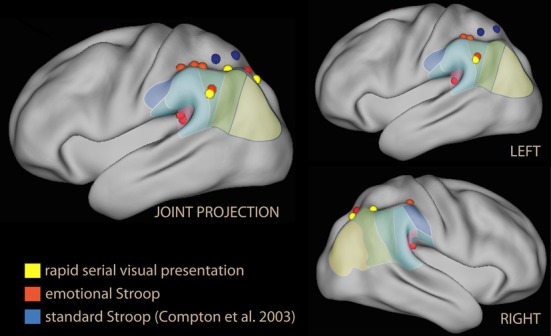
**Lateral rendering of foci from studies investigating emotional Stroop (in red), standard Stroop (in blue, from Compton et al., 2003), and rapid serial visual presentations of emotional distracters (in yellow)**.

The most obvious use of emotional material is as a spatial distracter in a selection or identification task (Eriksen and Eriksen, 1974). These studies show that emotional material as a distracter does not *per se* activate the IPL (Vuilleumier et al., 2001; Ochsner et al., 2009b). However, recruitment of the IPL was reported by studies where emotional stimuli were presented as temporally dislocated distracters (Figure 3, in yellow). Temporal distracters have an interfering effect on reports on the identity of a stimulus if they immediately precede or follow the target stimulus (Broadbent and Broadbent, 1987). In a study using a distracting non-informative word displayed briefly prior to the target word in a rapid serial presentation (Luo et al., 2007) the emotional and the sensory salience (supra- and subliminal) was varied independently. Increasing interference from distracters from subliminal to supraliminal increased activation in the SPL, while increasing interference by adding emotional valence increased activation in the IPL in supraliminal presentations. In this study, the effect of valence in the right IPL was the largest in the contrast.

Of particular relevance for the present issue is also the study by Mitchell et al. (2008), where emotional distracters of differing valence preceded and followed the target of a simple discrimination task. The IPL was activated together with the SPL by the task, but only the IPL was shown to interact with the presence or absence of emotional content in the distracters. *Post-hoc* analysis of this interaction showed that when distracter images were shown without the target, activity for negative distracters was highest and positive distracters lowest, while during the task the effect of valence was reversed. This suggests that institution of a task set resulted in different reactivity of the IPL to specific kind of distracter images, leading to suppression of the negative valence signal. Furthermore, activity in the IPL during the task was negatively correlated with amygdala reactivity to emotional valence. In this study, the only effect in the interaction was observed in the IPL. This study is consistent with the model of a filtering function of IPL for selecting relevant stimuli modified by the task set, showing that emotional stimuli are particularly effective in eliciting variations in the activity of the region. The findings are also consistent with the dissociation between this level of processing of emotional stimuli and the sensory encoding taking place in the amygdala.

**Figure 4:**
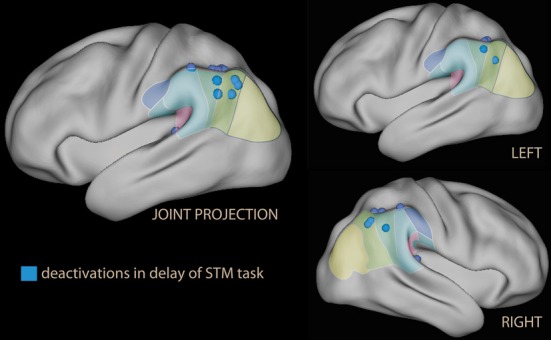
**Lateral rendering of foci of relative deactivation brought about by emotional stimuli used as distracters in the delay phase of a short-term memory (STM) task**.

In other studies where emotional stimuli appeared during the retention interval of a short-term memory task, however, no effect of emotion in the IPL was reported (Dolcos and McCarthy, 2006). Instead, these studies consistently reported relative deactivation of the IPL with emotional material (deactivation or less activation than in the neutral condition; Dolcos and McCarthy, 2006; Dolcos et al., 2008; Chuah et al., 2010; Denkova et al., 2010; Oei et al., 2011; Iordan et al., 2013; see blue foci of Figure 4).

The second group of studies used emotional tone as a task-irrelevant dimension of the target. Among the first studies in this group were those investigating the emotional Stroop. In the standard Stroop, the correct choice in the task and the distracting feature in the stimulus interfere directly. This task elicits strong activation in regions associated with attention and working memory (Figure 1A), consistent with the need to maintain task-relevant information online, exclude distracting features of the stimulus, and override a prepotent response tendency (Pardo et al., 1990; Banich et al., 2000). In the emotional Stroop, in contrast, the emotional tone of the stimulus may be a distracter only in virtue of its salience, and there is no direct conflict with response (Algom et al., 2004); accordingly, the interference-related activations in dorsal control areas are smaller (George et al., 1994). Whalen et al. (1998a) reported no effect on the IPL by emotional stimuli, but a dissociation between the ventral and dorsal anteromedial prefrontal cortex, accompanied by an activation/deactivation dissociation in the main task. Also Shafer et al. (2012) reported no IPL effect of emotion in a task with many elements in common with the emotional Stroop. The finding differed in the study by Compton et al. (2003), which explicitly focused on demonstrating the existence of an emotional/cognitive dissociation in the parietal lobes, and distinguished between the effects of valence and arousal in the stimuli. They found large activations in the superior parietal lobule for the standard Stroop (Figure 3, in blue). In contrast, none of the emotional conditions increased activity here; instead, negative emotion was associated with increased activity in the IPL bilaterally and in the left supramarginal gyrus, which was driven by valence (Figure 3, in red). However, the study did not report whether the dorsal/ventral dissociation was accompanied by analogous activation/deactivation dissociation in the main task.

Viewed together, the studies of this section report modulation of IPL by emotional distracters that depends on the relationship between the presence of emotional tone and the control processes activated by the task. When emotional tone was added to a distracter whose encoding was forced by the psychophysical properties of the presentation, as in the rapid serial presentation task, IPL was more active than with neutral distracters. In contrast, when emotional tone was added to distracters that could be effectively excluded by input processing, as in the retention interval of a short-memory task, IPL was more deactivated by emotional than by neutral stimuli. However, there were also studies in which IPL did not seem to be modulated by emotion.

### Cognitive/motor inhibition

These studies are characterized by conflict in the generation of response due to an automatic association between some aspects of the stimulus set and the wrong response. Automaticity here ensues from an overlearned response in association with the stimulus that gives rise to the conflict (Logan, 1988), or to the rapid instantiation of a response habit in the presence of a large number of trials where the correct response is always the same. In this setting, the overlearned response must be inhibited for correct task execution. Studies in this group investigate the effect of emotional tone in combination with response inhibition.

**Figure 5:**
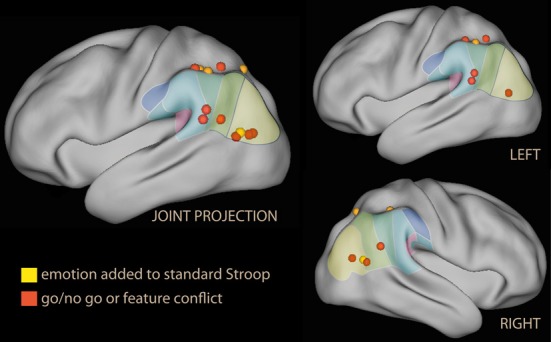
**Lateral rendering of foci from studies investigating the effect of emotional distracters in tasks with prepotent response or motor inhibition**.

A typical representative of this kind of study is the go/no go paradigm. Here, a stimulus requiring a response occurs frequently, while a rarely occurring stimulus requires no response. Go/no go studies elicit activation in a complex network associated not only with allocation of attention, but also with the necessity to regulate a prepotent motor response. Several studies have associated distinct prefrontal regions with the inhibitory component (Aron et al., 2004; Chambers et al., 2009; Sharp et al., 2010). In the present review, the focus is on the ventral parietal regions and its possible association with the attentional component of the task. In go/no-go studies no activation of the IPL was observed when the criterion determining the go or no-go response was the presence of emotional valence itself (Elliott et al., 2000). However, when emotional valence connotated stimuli incidentally, modulation of several cortical regions was observed, the most prominent of which were the ventromedial prefrontal cortex/orbitofrontal cortex, IFG, and right IPL (Goldstein et al., 2007; Brown et al., 2012; see Figure 5, red). IPL recruitment was also reported by studies adding emotional tone in the context of the conflict engendered by the standard Stroop (Krebs et al., 2011).

Another group of studies introduced a conflict between the emotional tone and another aspect of the stimulus by superimposing faces and conflicting or congruent written text. These studies reported no consistent effect in the IPL (Table 1).

As in the spatial attention studies, activation of IPL in studies of this group seems to be favored by the lack of an informativeness of emotional tone for the task at hand.

### Subliminal presentation of emotional stimuli

The study by Luo et al. (2007) suggests that the effect of emotional valence of stimuli on the IPL requires supraliminal exposure, in contrast with the effect on the amygdala (Morris et al., 1999; Whalen et al., 2004; de Gelder et al., 2005; Liddell et al., 2005; Vuilleumier, 2005). If this is correct, then we should not observe any IPL effect (which might conceivably be associated with covert orienting) in studies of subliminal exposure to emotional material. To verify this hypothesis, neuroimaging studies where faces bearing an emotional expression were presented subliminally were examined to see if they reported IPL effects (Table 2). The findings of this survey were not consistent. Even if the majority of studies reported no effects in the IPL, two studies did. However, in the study by Phillips et al. (2004), effects were present for both subliminal and supraliminal presentations, although differently lateralized; furthermore, the supraliminal effects were larger. On both studies, the test statistic was well below thresholds required by multiple comparison corrections; this is in contrast with the magnitude of effects in the studies of the previous section, which were at times the strongest across the brain. One may conclude that there is no strong evidence for an effect in IPL at the presentation of subliminal emotional stimuli.

**Table 2:** **Effects on IPL of subliminal presentation of faces with emotional expression**.

**Study**	**Notes**	**Exposure (ms)**	**IPL effect**
Whalen et al., 1998b		33	No
Morris et al., 1999	Conditioned and non-conditioned stimuli	30	No
Killgore and Yurgelun-Todd, 2004		20	No
Luo et al., 2004		30	No
Nomura et al., 2004		35	No
Phillips et al., 2004	Both sub- and supraliminal; larger effects in supra	30	Yes
Etkin et al., 2004		33	Yes
Liddell et al., 2005		16.7	No
Harmer et al., 2006		17	No

For criteria of inclusion, see the Methods section.

### Declarative memory of emotional stimuli

While in many respect different from studies of attention, studies of memory are included here because of the evidence for a dissociation between dorsal and ventral left parietal areas with many aspects in common with the dissociation demonstrated by studies of attention (Cabeza et al., 2008, 2012; Ciaramelli et al., 2008; Uncapher and Wagner, 2009). A possible connection between these two types of studies may be seen by viewing declarative memory tasks as selection from a set of internal representations.

At behavioral level, the influence of emotion on memory processes is shown by enhanced accuracy and vividness of declarative memories (Kensinger, 2004). These effects may arise from effect of emotion at different stages of the memory process, from encoding to consolidation and retrieval (LaBar and Cabeza, 2006). As in the modulation of attention by emotion, the amygdala is thought to interact with prefrontal function to fine-tune memory to emotional content (LaBar and Cabeza, 2006).

Neuroimaging studies have investigated the impact of emotion on memory much more systematically than in orienting paradigms of the previous section. A recent meta-analysis evaluated results from 15 carefully selected neuroimaging studies on successful emotional memory encoding (Murty et al., 2010). Of interest in the present context is the involvement of ventral parietal areas in emotional memory paradigms, beside the well-known involvement of amygdala, medial temporal, and prefrontal areas. Murty et al. (2010) found a significant effect in the right IPL/supramarginal gyrus associated with successful encoding of emotional relative to neutral stimuli. Commenting on the contrast with the result of the systematic review by Uncapher and Wagner (2009), where activation in this area was associated with inferior memory performance, Murty and colleagues conjectured that recruitment of the reflexive orienting process associated with IPL by emotional material might have been representative of the beneficial effects of reflexive orienting in an ecologically complex setting.

### Summary on neuroimaging studies of attention to emotion

An effect of emotion on IPL was reported by about 65% of the reviewed studies of attention to emotion. Localization was on the right or indifferent in about 50% of studies; in all cases where localization was on the left, distracters were verbal. In 15% of the studies IPL activation was the largest reported effect in the contrast. However, the involvement of IPL was complex, as some studies reported its deactivation by emotion, in contrast with the majority of findings. This may be due to considerable diversity of the studies examined here. In several cases, however, it appears that these heterogeneous findings were influenced by the relationship between the stimuli and the task set.

In the studies of spatial attention, for example, where emotional tone was added to a spatial cue, activation in the IPL was reported when the cue was not informative for detecting the target (Armony and Dolan, 2002; Pourtois et al., 2006), in contrast with the effect of cues that informed about the location of its appearance (Tosoni et al., 2013). In studies with emotional distracters, where the distracters were spatially distinct from the target and were presumably inhibited by selection, no activation in IPL was detected. However, strong IPL activations were reported by studies in which the emotional distracter immediately preceded the target at the fixation center (rapid serial presentation tasks: Luo et al., 2007; Mitchell et al., 2008). These differing results may be due to the fact that in rapid serial presentation tasks processing of the distracter cannot be avoided, as it is shown by the interference in identifying the target (Broadbent and Broadbent, 1987).

No consistent activation of the IPL was reported by studies where the emotional distracter and the target were combined in the same stimulus or were spatially superimposed. Similarly, no consistent evidence for a role of IPL was provided by studies of conflict in the generation of response, such as the go/no go or standard Stroop. This may be due to the fact that the nature of the conflict here is on the side of response, not perception. A striking exception to this pattern is given by the studies by Goldstein et al. (2007), Krebs et al. (2011), and Brown et al. (2012). In all these studies, emotional tone was added to stimulus material that was used in the task generating response conflict. The presence of absence of emotional tone was not informative to make the decision, and perceptual encoding of the material was essential to the task. Here, activation of the IPL was robust.

It therefore appears that activation of the IPL by emotional distracters was contingent on the task set, additionally modulated by the locus of interference. When interference was on the perceptual side between stimuli, little activation in the IPL was seen unless processing of emotional stimuli was forced by the rapid serial visual presentation task. In studies where interference was on the response side, activation of the IPL was seen where emotional tone incidentally connoted stimuli used in the task, as in the studies by Goldstein et al. (2007), Krebs et al. (2011), and Brown et al. (2012).

These activations are consistent with a recruitment of IPL by emotional material but, considered in isolation, do not tell us unequivocally if they were due to increased top-down control in the presence of emotional distracters, to increased interference, or to attentional capture as in ventral network reorienting. Nevertheless, their ventral localization, and the tendency to occur when the emotional tone was not informative for the task, do not suggest direct involvement of top-down suppression of distracters. However, another aspect of these data speaks more decisively against interpreting IPL activation as the correlate of top-down control or increased interference, as activations of DLPFC may be. This is given by the studies of short-term memory or working memory in which emotion was associated with IPL deactivations (see Dolcos and McCarthy, 2006 and the analogous studies in Table 1). If IPL were the neural substrate of top-down control like SPL or DLPFC, we would expect it to be always activated—or at least not deactivated—by content designed to increase interference. In contrast, these deactivations parallel those of studies of cognition reporting an association between proactive control and deactivation of the ventral attentional network (Todd et al., 2005; Shulman et al., 2007). IPL activations may be observed when the emotional tone of distracters is embedded within the task so as to escape proactive control, as when it is not informative for the task, or when its encoding is essential for the task but the focus of control is on response. Because detected in comparison with neutral stimuli, the deactivations suggest that proactive control on the emotional features, brought about by their relevance to the task, may modulate the activation level in the IPL more strongly than non-emotional features of the stimuli. This conclusion is consistent with data from the effect of emotional tone on IPL recruitment in studies of declarative memory of emotion reviewed by Murty et al. (2010).

In interpreting these data, it is useful to remember that all these results emerged by contrasts between emotional and neutral distracters, which modeled at the second level the interaction between the task and emotional tone. They provide evidence consistent with the notion that emotional material may preferentially trigger activation in ventral parietal areas, as expected from stimuli of high behavioral relevance. In models developed in studies of non-human primates, the role of anterior parietal cortex is the computation of the relative behavioral relevance of stimuli to guide choice or selection on the basis of information from locations in extrapersonal space (Itti and Koch, 2001; Gottlieb, 2006; Andersen and Cui, 2009; Bisley and Goldberg, 2010; Freedman and Assad, 2011). To the extent that these ventral areas contributed to determining priorities in the handling of information in the studies reviewed here, they may also have contributed to prioritizing emotional information in specific task set and interference configurations.

There is also some indication that valence, especially negative valence, was more important to elicit the IPL effect than arousal levels (Compton et al., 2003). Emotional arousal is associated with activation of the amygdala (Whalen et al., 2002). The review of IPL effects in studies of subliminal emotional stimulation also suggests that robust effects of emotional tone in the IPL require stimuli to be presented supraliminally, in contrast with findings in the amygdala. This differentiates emotional processing in the IPL and in the amygdala.

A possible limitation of the present attempt to summarize results is the localization of many foci near the IPS. Because some degree of heterogeneity in collating data from different studies is inevitable, it is possible that some of the foci attributed to the effect of emotional intensity were located in the IPS, especially those situated more dorsally. These foci may then more appropriately be considered a correlate of activity of the dorsal network system, perhaps as a result of increasing interference from the emotional distracters. Discussion of this issue will be postponed to after considering data from emotion regulation studies.

Notwithstanding its common occurrence, the involvement of the IPL is not mentioned very often in the discussion of findings. This is remarkable since in some studies IPL involvement was quantitatively the most extensive or the most intense in the contrast opposing emotional and neutral stimuli (Compton et al., 2003; Goldstein et al., 2007; Luo et al., 2007; Mitchell et al., 2008). A similar remark was made by Murty et al. (2010) in the discussion of their meta-analysis of effects of emotion on parietal areas in studies of declarative memory. This relative neglect of ventral parietal involvement may depend on a hypothesis-driven focus on prefrontal regions as the substrate of cognitive control processes.

While these data provide some support to the notion that emotional tone may be part of the “behavioral relevance” category that preferentially triggers ventral network reorienting, they do not inform us on the relevance of the ventral network in emotion regulation. Studies of emotion regulation will be examined in the next section.

## Neuroimaging studies of voluntary emotion regulation

In studies of voluntary emotion regulation, participants are instructed to execute a specific strategy to change their emotional reaction. Usually, but not always, the strategy involves down-regulating the reaction to an emotional stimulus (most often but not invariably negative). Strategies vary between studies, including simple suppression of erotic arousal (Beauregard et al., 2001), self-distraction (Kalisch et al., 2006), distraction by execution of a demanding cognitive task (Kanske et al., 2011), and suppressing expression of emotion vs. using cognitive reappraisal (Ochsner et al., 2002, 2004; Goldin et al., 2008). When used to down-regulate emotion, cognitive reappraisal is the recontextualization or reframing of a negative stimulus in less negative terms (Ochsner and Gross, 2008). This latter strategy is the most intensively investigated in recent studies.

Studies of emotion regulation are characterized by the instruction to change one's own affective state. This is in contrast with the attention to emotion studies of the previous section, in which the instruction referred to a cognitive task that remained the same, while experimental variation was introduced by adding emotional tone to stimuli. A well-known finding of about all voluntary emotion regulation studies is recruitment of the dorsal prefrontal cortex in both medial and lateral aspects, complemented by activation in the VLPFC/IFG (Ochsner et al., 2012). Therefore, the evidence for the involvement of the prefrontal portion of the dorsal attentional network in voluntary emotion regulation is overwhelmingly strong. This evidence constitutes the empirical support for the dual-process view of the mechanisms underlying cognitive emotion regulation (Ochsner and Gross, 2008). The findings on the parietal lobe, divided by dorsal and ventral localization, are summarized in Table 3.

**Table 3:** **Effects on SPL and IPL of voluntary emotion regulation**.

**Study**	**Task**	**SPL/IPS effect**	**IPL effect**
Beauregard et al., 2001	Allowing or inhibiting sexual arousal while watching erotic movies	Yes	No
Ochsner et al., 2002	Reappraise or watch negative scenes	No	Yes +
Lévesque et al., 2003	Suppress or allow reaction to sad movie	No	No
Ochsner et al., 2004	Reappraise or watch negative or neutral images	No	Yes
Phan et al., 2004	Reappraise or maintain impression aversive scenes	No	No
Phan et al., 2005	Suppress or maintain, negative or fixation	No	No
Harenski and Hamann, 2006	Pretend scene unreal or view of moral violations	No	No
Kalisch et al., 2006	Think of something else or allow influence of anticipation of pain	No	No
Ohira et al., 2006	Suppress emotional response or attend negative, neutral, or positive images	Yes	No
Urry et al., 2006	Increase, decrease or maintain negative picture stimuli	No	No
Kim and Hamann, 2007	Increase, decrease, or left unaltered the influence of positive or negative emotions	No	No
Herwig et al., 2007	“Reality check” of psychotherapy or no instruction, anticipation of negative, positive, or unknown images	No	No
Eippert et al., 2007	Consider as not real or view, negative of neutral	No	No
Johnstone et al., 2007	Increase/decrease or maintain, negative or positive	No	No
Delgado et al., 2008	React normally or think of something calming when viewing aversively conditioned cue	No	No
Goldin et al., 2008	Reappraise, suppress, or watch negative or neutral images	No	Yes
Wager et al., 2008	Reappraise or watch, negative or neutral	No	Yes
McRae et al., 2008	Reappraise or watch, negative or neutral	No	Yes
Koenigsberg et al., 2009	Self-distance or attend, negative or neutral	Yes +	Yes
Mak et al., 2009	Unspecified reduction negative emotion or view, negative, positive, or neutral images	No	No
New et al., 2009	Reappraise, enhance, or maintain, negative or neutral	Yes	No
Sheline et al., 2009	Look or “reframe picture context,” negative or neutral	No	No
Staudinger et al., 2009	Self-distance or allow emotion while gambling	No	Yes +
Urry et al., 2009	Increase, decrease, or maintain of negative images	No	No
Modinos et al., 2010	Reappraise or look, negative or neutral	No	Yes +
Hayes et al., 2010	Reappraise, suppress, or view, negative or neutral images	Yes	Yes
Domes et al., 2010	View as not real, increase, or maintain, negative or neutral	No	Yes
Walter et al., 2010	Priming reappraisal prior to watching negative stimuli	No	Yes
Erk et al., 2010	Reappraise or look tested on a delayed presentation of negative or neutral stimulus	No	Yes +
Koenigsberg et al., 2010	Self-distance or attend, negative or neutral images	Yes	Yes
Schardt et al., 2010	Detach or look, negative or neutral images	Yes	Yes +
McRae et al., 2010	Reappraise or look, negative or neutral	No	Yes
Staudinger et al., 2011	Reappraise or allow, reward anticipation	No	Yes +
Winecoff et al., 2011	Detach or experience, negative, neutral, or positive images	Yes	Yes
Kanske et al., 2011	Reappraise/dual task or view, negative or neutral	No	Yes +
Hutcherson et al., 2012	Distance, allow, or enhance desire for food in fasting participants	No	Yes
Krendl et al., 2012	Decrease or attend, negative or positive	No	No
McRae et al., 2012a	Reappraise or watch, negative or neutral	No	Yes

Data refer to the contrast regulate vs. look. Yes +: the plus sign indicates that the IPL effect was the strongest in the contrast. The word “reappraise” was used for studies that cite Ochsner et al. (2002) or later work to instruct participant on reappraisal; “increase/decrease or maintain” denote studies that refer to Jackson et al. (2000) or Jackson et al. (2003) for participant instruction. For criteria of selection of studies in the table, see the Methods section.

**Figure 6:**
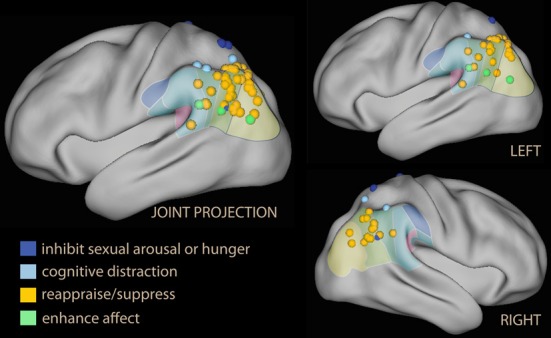
**Lateral projection of foci detected in the parietal lobe and the temporo-parietal junction in studies of voluntary emotion regulation**. In blue, studies using block or inhibition of sexual arousal or hunger; in light blue, foci from parietal activation while distracting by executing a concomitant task. Studies of reappraisal, suppression, or self-detachment are in orange; in green are the foci from the few studies in which the instruction was to enhance, not suppress, emotional reaction.

Inspection of this table reveals that the IPL is more often recruited than SPL by voluntary emotion regulation (logistic regression with repeated measurements, *z* = 2.9, *p* = 0.003). Furthermore, there is a tendency for the studies showing no IPL recruitment to have been carried out earlier, when instructions simply to suppress one's reaction to the stimulus were common (Beauregard et al., 2001; Lévesque et al., 2003; Kalisch et al., 2006; Ohira et al., 2006; Kim and Hamann, 2007; logistic regression on time of publication, *z* = 2.5, *p* = 0.01). Some studies reporting foci in the SPL instructions to inhibit sexual arousal. In Figure 6, these foci were shown in blue, while foci detected by reappraise, suppress, or self-detach instructions are in orange. Foci reported while instructing to enhance the reaction to emotional stimuli are in light green. Studies reporting on enhancement instructions are few, and the reported foci do not appear to deviate systematically from those detected with reappraise or suppress instructions.

An issue raised by this finding is the status of some foci located in the superior part of the IPL, which may originate in the IPS and for this reason may be considered part of the dorsal network (in Table 3, Koenigsberg et al., 2009, 2010; New et al., 2009). However, if one looks at the IPL clusters in studies that displayed the effect on a lateral surface rendering, it becomes apparent that they extended toward the temporal lobe, usually including TPJ (Ochsner et al., 2004; McRae et al., 2008; Domes et al., 2010; Erk et al., 2010; Modinos et al., 2010; Staudinger et al., 2011; McRae et al., 2012a). Only one study (New et al., 2009) shows an effect located within and limited to the IPS.

Another finding was that the peak effect on IPL was in many studies the strongest in the contrast with the look instruction, suggesting that the effect in the parietal lobe was more marked than in the prefrontal cortex. These studies are marked by “Yes +” in Table 3. In several studies (Ochsner et al., 2002; Wager et al., 2008; Drabant et al., 2009), the IPL peak correlated with self-reported efficacy of emotion regulation. Interestingly, IPL also appears to be the region that best differentiated the neural correlates of reappraisal in borderline personality relative to healthy controls (Schulze et al., 2011; Lang et al., 2012; see also Koenigsberg et al., 2009). In a recent study on the effects of psychotherapy of social anxiety assessed with a cognitive reappraisal probe, the interaction between time before and after therapy and the down-regulation instruction localized in the IPL (Goldin et al., 2013).

In conclusion, studies of voluntary emotion regulation (particularly those relying on cognitive reappraisal) appear to recruit the dorsal attentional network in the prefrontal lobes, but activate the IPL, the hub of the ventral attentional network, in the parietal lobes. Notwithstanding its striking prominence, IPL effects are not referred to often in these studies, and have only recently been explicitly noticed (Ochsner et al., 2012). Instead, the interpretive framework of these studies focuses on the effect in the prefrontal areas, consistently with the dual-process view of emotion regulation. A few key studies, however, offer possible insights on the interpretation of this pattern of anterior/posterior dissociation.

In the studies by McRae et al. (2010) and Kanske et al. (2011), activations elicited by cognitive reappraisal were compared with those detected in a “distraction” condition, which consisted in the simultaneous execution of a short-term memory (McRae et al., 2010) or a demanding working memory task (Kanske et al., 2011). Starting from the seminal findings by Hariri et al. (2000) and Liberzon et al. (2000), many neuroimaging studies have shown that attentional engagement in demanding tasks affects the sensory encoding of emotional stimuli in the limbic system and the amygdala, reducing the activation that may be attributed to emotional arousal (Pessoa, 2008). In the studies by Kanske and McRae, the concurrent cognitive task activated the SPL or the IPS (in light blue in Figure 6), while the activation of the cognitive reappraisal condition was shifted to the IPL, with a narrow area of overlap centered on the IPS. In Kanske et al. (2011) most of the activation in the dorsal prefrontal areas was shared between the cognitive reappraisal and the concurrent cognitive task, but cognitive reappraisal additionally recruited the ventrolateral prefrontal cortex. In McRae et al. (2010) the reappraisal task recruited additional ventrolateral and antero-medial prefrontal areas. On the basis of these data, therefore, an argument may be made that an important and distinctive neural correlate of cognitive reappraisal (as opposed to turning attention elsewhere, or just blocking affect) lies in the ventral, not the dorsal, activations associated with this task. Following further this line of reasoning, one may distinguish between relatively non-specific effects of attentional recruitment on the control of emotional arousal, common to all strategies of voluntary control and associated with prefrontal areas related to working memory and the dorsal network, and contributions from ventral network areas active during cognitive reappraisal. As noted in the discussion of the attention to emotion section, it is difficult to interpret activation in the ventral IPL as a correlate of top-down control, as these same areas were deactivated in studies requiring suppression of emotional distracters.

## Thought control and spontaneous avoidance of negative material

While emotional content has in most respects a facilitatory effect on attentional processes, there are specific cases where it is also known to slow down processing or induce avoidance, especially in the context of stimuli that are aversive or endowed with negative valence (Gray et al., 2002; Sagaspe et al., 2011). Of particular importance to the understanding of mood disorders is the avoidance of negative cognitions in the healthy, in contrast to what is observed in depressed individuals (Beck, 1976). The bias for positive cognitions is empirically demonstrable with the scrambled sentences task (SST, Wenzlaff, 1991). Participants are presented with a set of scrambled words from which they can assemble one of two possible sentences, depending on the order and the selection of words from the set. When the two alternative sentences have pessimistic and optimistic connotations, healthy participants spontaneously avoid the pessimistic alternative even if no reference to the valence of the sentence was given in the instruction (for example, the set “is bleak the future bright” can be recomposed in either “the future is bright” or “the future is bleak”). This bias is associated with the absence of previous depressive episodes or symptoms and predicts future episodes (Rude et al., 2002, 2003, 2010; Wenzlaff et al., 2002). It also correlates with depression scores or assessment of mood (Rude et al., 2010; Viviani et al., 2010).

While relevant to assess the tendency to negative cognitions of depression, the roots of the SST are in a highly developed cognitive model of how the control of thoughts through executive attentional processes is achieved or may fail (Wenzlaff et al., 1988; Wegner et al., 1993; Wegner, 1994). According to this model two processes, differing in the amount of resources they require, work together to promote desired mental states. A “monitoring process,” not very resource demanding, is continuously active in the background to detect the emergence of undesired content. When this happens, the monitoring process triggers an “operating process,” much more resource demanding, to attend to and suppress the undesired content. The operating process acts therefore like an executive process down-regulating negative emotional content in models of cognitive control of emotion. There are also similarities between the monitoring process and the concept of “bottom-up attention” to internal memories of ventral network theorists. In both cases, these processes are associated with endogenous ideas competing for inclusion in working memory, and have a potentially disruptive effect. However, the notion of monitoring process is explicitly linked to desires for mental states in the definition of the kind of salience that triggers it. According to the thought control model, avoidance of negative words in the chosen sentences in the SST is initiated spontaneously through this mechanism.

The SST is therefore of interest as a paradigm that, according to the model that inspired it, triggers a regulatory process without the influence of an explicit instruction to regulate, in contrast to voluntary emotion regulation studies. If avoidance of negative thoughts in the SST is obtained by a control process of executive nature, we should observe recruitment of the dorsal network, or of the part of it that executes this control at the net of the effect of the instruction of the experimenter. This is a prediction not only of the thought control model, but also of dual-process models of control, because negative words are commonly more salient than positive words (Bradley and Lang, 1994), therefore requiring more top-down control to be suppressed. A second issue raised by the SST is the neural correlate of the monitoring process, and the recruitment of ventral attentional areas that might conceivably support the parallelism between the “monitoring process” and “bottom-up” attention.

**Figure 7:**
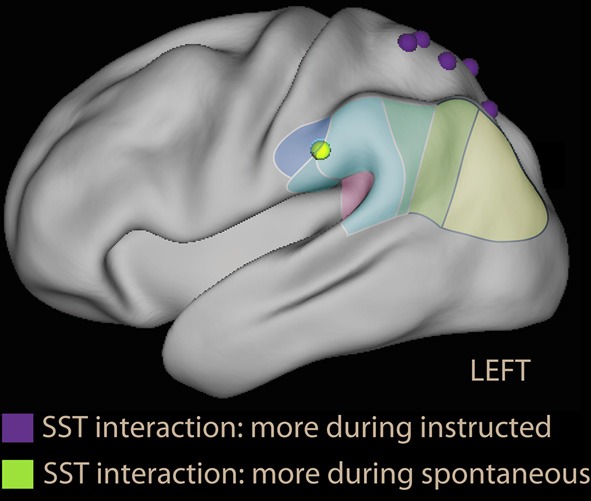
**Parietal foci of interaction instruction × emotion from the scrambled sentences task study (Viviani et al., 2010)**. In violet are foci that were more active during the instructed avoidance of negative sentences; in green the focus more active during spontaneous formation of sentences in the supramarginal gyrus. Note that the relative activation in the supramarginal gyrus is located more anteriorly than in voluntary emotion regulation studies, and is more similarly distributed to the ventral foci of studies with covert orienting to reward (Armony and Dolan, 2002, Figure 2) and emotional Stroop foci (Figure 3).

A neuroimaging study of thought control based on giving explicit instructions to participants found activation in medial dorsal areas, but no activity in DLPFC or SPL; instead, activity was modulated in the insula and IPL (Wyland et al., 2003). The study by Viviani et al. (2010) used the SST to identify the areas activated while avoiding negative content in the absence of an explicit instruction. Two factors were present in the study: emotional and neutral sentences (to control for sentence selection) and making no mention of the emotional content vs. asking participants explicitly to avoid the pessimistic alternative (to compare spontaneous and instructed avoidance). The main finding was in contrast with the prediction of the thought control model. In the spontaneous group, the dorsal attentional network was not recruited by the emotional sentences; on the contrary, the dorsal attentional network was less recruited than when sentences were neutral. In contrast, in the instructed group a small increase in activation in the DLPFC was seen in the group explicitly instructed to avoid the negative alternative. Importantly, a significant emotion × instruction interaction was observed (led by the decrease of activation in DLPFC in the spontaneous group). Hence, the effects on the dorsal attentional network could not be explained by the presence of emotional material or the absence of an explicit instruction alone. In the parietal lobe, the interaction showed prevalent recruitment of dorsal areas in the instructed, and ventral areas in the spontaneous group when confronted with emotional material (Figure 7, foci in violet and green). Similar effects were observed in the medial prefrontal cortex and the posterior cingulus.

This study used an arterial spin labeling technique to identify areas activated and deactivated by the sentence-forming task. Comparison of the contrast task vs. baseline and the areas identified by the interaction showed that the focus in the anterior IPL, in the prefrontal cortex and the posterior cingulus were deactivated in the sentence forming task. The increase of IPL perfusion in the spontaneous group when exposed to emotional sentences took place within the context of a task deactivation. In contrast, in the instructed group the presence of an explicit instruction to avoid negative sentences was associated with similar deactivations in the neutral and the emotional material.

A second study on the tendency to use emotional words provided indirect evidence for a modulation of areas deactivated by the task consistent with a ventral network model (Benelli et al., 2012). In this study, participants were asked to read short textual descriptions of a scene rich with potential emotional issues. Textual descriptions varied systematically along two factors: presence or absence of emotional words, and of abstract words. The key aspect of the study was that after the scan participants were asked to give their own written account of what happened in the scene they had viewed. The account was then scored for the use of emotional or abstract words. Individual differences in the propensity to use or avoid emotional terms in the account were, as in the previous studies, related to spontaneous tendencies to avoid emotional material, since there was no explicit instruction in this respect. These after-scan scores were then regressed on the contrasts from the two factors characterizing the textual descriptions participants were reading during the scan. Individual differences in the use (or avoidance) of emotional words was significantly associated with the effect of emotional material while reading textual descriptions. Importantly, these differences showed modulation of areas deactivated by the task relative to fixation, including modulation of the IPL, while there was no correlation between the tendency to avoid emotional material and the use of prefrontal areas activated by the task and that are associated with working memory processes.

These data are consistent with the notion that avoidance of negative but arousing words in the healthy may not depend on successful executive control of emotional stimuli. Instead, avoidance of negative words in the healthy may take place naturally and without particular effort, notwithstanding their salience. This finding is of interest also because it holds in prospect the possibility to investigate empirically “automatic” forms of emotion regulation with these or similar paradigms. The hypothesis, advanced in Viviani et al. (2010) that this avoidance may be related to ventral network orienting, in alternative to executive processes associated with the dorsal network, depends not only on the anatomical localization of the areas associated with spontaneous avoidance, but also on the evidence that ventral network orienting operates to allocate attention to items that are behaviorally relevant irrespective of both salience and the focus of endogenous attention. Furthermore, the prevalent deactivation of these areas in the contrast task vs. baseline in these studies is inconsistent with a classic top-down control process, but is consistent with the observed deactivations in the ventral attentional network, enhanced by proactive control on these semantic/secondary association areas when avoidance was voluntary.

## Discussion and perspectives for future research

The present review has been motivated by the contrast between theories highlighting the existence of a ventral network and those adopted by studies of attention to emotion and emotion regulation. These latter employ a dual-process model to interpret their data based on the opposition between sensory salience of stimuli competing for attention and top-down bias to influence the outcome of this competition. The evidence from ventral network studies suggests the existence of at least a third distinct brain circuit-cognitive process involved in attending to external stimuli (Corbetta et al., 2008) or to internal representations (Cabeza et al., 2008). This evidence shows that attention may be driven to information that is somehow “behaviorally relevant” even if neither salient nor currently targeted by top-down control, in contrast with the dual-process model.

A first issue of interest was the evidence in the review for the capacity of emotional information to trigger activation of the inferior parietal lobule (IPL), a key hub of the ventral network, which would speak for including emotional information in the category “behaviorally relevant” considered in spatial attention studies. All types of studies considered in the review provided evidence of IPL recruitment in the presence of emotional stimuli, albeit with different degrees of consistency. The strongest evidence was provided by studies of emotion regulation, where the IPL was much more often activated than its dorsal counterpart. However, also studies of attention to emotion and memory provided considerable evidence of IPL activation in the presence of emotional stimuli, especially considering those cases where this activation was the strongest in the brain. Furthermore, the few studies of attention to emotion that examined the effect of valence and arousal separately suggest that valence, especially negative valence, rather than emotional arousal, was specifically responsible for ventral network activity. This finding would be consistent with a distinction between limbic circuits involved in emotion processing, which are activated in a pre-attentive and relatively automatic fashion by emotional salience and mediate emotional arousal, and the ventral network, preferentially activated by valence (Brosch et al., 2011) during semantic encoding of stimuli.

A second issue was what these studies could reveal about the function of these areas in emotional processing, and the applicability of the ventral network model of attentional reorienting to characterize this function. Here, the diverse types of studies considered in the review provided information from different angles but, as it will be discussed here, also broadly consistent with ventral parietal areas being concerned with computing the priority of stimuli for the generation of response, as in ventral network reorienting. This account of IPL function must explain its modulation in attention to emotion tasks, together with SPL recruitment; the preferential recruitment of IPL, instead of SPL, in emotion regulation studies; and the modulation of IPL in the tasks without explicit instruction to regulate, as well as the lack of SPL or prefrontal recruitment in these latter paradigms, in contrast with instructed paradigms.

The modulation of IPL signal in studies of attention to emotion appeared to be complex and to be influenced by the interaction of the locus of interference and the task set. These complex effects suggest that a careful analysis of the form of load and locus of interference (Kahneman and Treisman, 1984; Harris and Pashler, 2004; Lavie et al., 2004; Okon-Singer et al., 2007) are required to properly interpret emotion to attention data. The most robust findings were IPL activations in studies where the emotional information had to be incidental to the criteria for the response required by the task, and interference was located on the side of the response. In contrast, in studies where the conflict was at the level of the stimulus, IPL activity was seen in circumstances that favored the perceptual processing of distracters. These findings suggest that IPL activity was observed when the emotional tone of the distracter was not as such the target of top-down control.

Importantly, in some studies where suppression of distracter was effective, as in the retention interval of a working memory task, the effect of emotional tone on IPL was the opposite, i.e., IPL was more strongly deactivated than with neutral distracter. This finding is difficult to reconcile with a simple top-down control role of IPL, as in this case we would expect IPL not to be deactivated by distracters. However, the observed deactivations are compatible with ventral network orienting. Deactivations in the ventral network have also been observed in spatial attention studies, where they have been interpreted as the neural correlate of a “filter” on incoming data associated with the existence of a task set (Todd et al., 2005; Shulman et al., 2007). One possibility is that these deactivations were the neural correlates of proactive control on potential distracters, acting on the late phases of sensory encoding, when the stimulus reaches semantic association areas. Within the framework of the bias competition model (Duncan and Miller, 2002), these deactivations may be the neural correlate of top-down control associated with task-specific adaptive coding of incoming stimuli. The stronger deactivation of IPL reported in studies where emotion was a source of interference to the task suggests that emotional stimuli might be particularly effective in eliciting such deactivations when control is active, either through their valence, or perhaps because of their semantic properties (Talmi and Moscovitch, 2004).

These findings may be best understood within a model of parietal function in which computation of priorities for response choice integrate not only executive goals and stimulus salience, but also a wider class of sources of information of behavioral relevance. Awh et al. (2012) have summarized data on the interaction between attentional processes and reward-conditioned stimuli or the past history of exposure to stimuli that, as discussed here for emotional stimuli, show that a simple dichotomy between bottom-up sensory salience and top-down control is insufficient to account fully for existent observations. Their proposal is the integration of information deriving from past experience on the stimuli to compute priorities for attentional allocation (Awh et al., 2012). These priorities may be represented in the IPS, from which they would be brought forward to prefrontal areas to be mapped to motor effectors (Andersen and Cui, 2009). In the emotion regulation studies by McRae et al. (2010) and Kanske et al. (2011) described above, distraction by a cognitive task and reappraisal shared activation in the IPS, but otherwise dissociated between DPL and IPL. These studies suggest that IPS may integrate priority information from sensory salience and top-down sources in dorsal areas (Vandenberghe et al., 2001; Yantis et al., 2002) and from behavioral relevance in ventral areas of the parietal lobes.

The notion that the parietal cortex contains priority maps integrating information of different nature receives support from neurophysiological studies in non-human primates. Although with different nuances, many researches stress that neurons in parietal areas dynamically compute abstract priority maps of stimuli in external space based on information of different nature (Platt and Glimcher, 1999; Gottlieb, 2006; Andersen and Cui, 2009; Kable and Glimcher, 2009; Bisley and Goldberg, 2010; Freedman and Assad, 2011). However, studies in non-human primates have described no dissociation between dorsal and ventral areas akin to the one considered here from neuroimaging studies in man. Nevertheless, the priority map model is consistent with the strong modulation of IPL activation by emotion observed in the reviewed neuroimaging studies, and with including emotional valence in the behaviorally relevant category that may activate the ventral network.

In these priority map models, the role of multimodal association areas may be not simply one of passive repository of semantic memory, but of actively contributing to computation of priorities for choice of response (Dorris and Glimcher, 2004; Kable and Glimcher, 2009; Freedman and Assad, 2011; Fitzgerald et al., 2013). This is consistent with the possibility that criteria other than sensory salience or effort may determine response, thus challenging the dual-process model. This possibility also challenges the “modal” view that control of response in the face of sensory salience is the prerogative of executive function (Kahneman and Treisman, 1984; Kahneman and Frederick, 2002). Representations of value in the IPL may confer priorities to stimuli and thus override sensory salience even in the absence of executive processes. In the scrambled sentences task, for example, assigning priorities between negative and positive words in the spatial array of the scrambled word set was associated with relative activation of anterior IPL when the choice of sentence was spontaneous (Viviani et al., 2010). In the presence of an explicit instruction, in contrast, the relative activation of IPL for emotional words was reduced. The role of IPL in computing priorities for response may also explain modulation of response to emotional information in paradigms without an explicit instruction on the form of this response, even without additional recruitment of prefrontal areas associated with top-down control.

The opposite of proactive control may be characterized as unconstrained reorienting to stimuli or, in an internal semantic space, forms of thinking that give precedence to spontaneously emerged representations. When production of thought is spontaneous, and no proactive control is in effect, we may accordingly expect activation of semantic association areas. In this respect, the ventral attentional network appears to be functionally similar to the default network system, explaining the apparent anatomical overlap. As it has been noted, the default network system co-localizes with semantic association areas (Binder et al., 2009), is active during spontaneous (Buckner and Carroll, 2007) or associative thinking (Bar, 2007; for a different view, see Sestieri et al., 2010). This form of cognitive process would have the properties attributed to “bottom up” attention by memory researchers (Cabeza et al., 2012), qualified by the exclusion of automatic reorienting to sensory salience. There are also considerable similarities between the properties of “bottom up” attention and the monitoring process of theories of thought control (Table 4).

**Table 4:** **Comparison of processes in dual-process, thought control, and the proposed orienting to behavioral relevance model of emotion regulation**.

**Process domain**	**Dual-process models**	**Thought control models**	**Proposed model**
Bottom-up automatic encoding, general	External stimuli compete for attention in the input channel	Endogenous ideas compete for inclusion in thoughts	External stimuli or endogenous ideas are represented in the late stages of long-term memory
Bottom-up automatic encoding, emotional material	Emotional stimuli possess specific salience properties giving them an advantage in competition with other inputs	Some endogenous ideas can be charged and tend to emerge	Emotional stimuli possess specific salience properties in both the input channel and in the ventral network
Intermediate processes, general	Not contemplated	Monitoring processes signal the emergence of ideas or thoughts but have low impact on limited capacity resources	Ventral network is triggered by stimuli that are relevant for the task at hand, or are behaviorally relevant, but is distinct from dorsal network
Intermediate processes, emotional material	Not contemplated	Monitoring processes can paradoxically keep emotional ideas active, for example in vulnerability to depression	Ventral network is triggered by long-term memory representations of individual salience, i.e. conditioned stimuli, or by emotional content, but may also apply own endogenous bias in semantic space as in healthy optimism (affective heuristics)
Executive processes, general	Top-down executive processes bias competition using limited capacity resources	Top-down executive processes set up monitoring processes and suppress endogenous ideas using limited capacity resources	Dorsal network biases competition directly or sets up ventral network to suppress/bias irrelevant stimuli
Executive processes, emotional material	Top-down executive processes and limited resources are taxed by need to bias against emotionally salient stimuli	Top-down executive processes are challenged by emotional content kept active by continuous monitoring	Dorsal network must handle triggers from ventral network produced by emotional stimuli
Failure of emotion regulation	Emotion dysregulation arises from increased reactivity to emotional stimuli and/or low top-down control capacity	Thought control difficulties arise when continuous monitoring has activated specific endogenous ideas	Emotion dysregulation may arise from dysfunction in the interplay between three processes, not two

This model may also explain why ventral activation in the left parietal lobes and TPJ is observed in studies of cognitive reappraisal. The cognitive reappraisal instruction contains an invitation for participants to imagine a favorable outcome or create a different interpretive framework for the presented emotional scene. This instruction requires participants to think original thoughts not determined strictly by stimuli or task. In contrast, studies where participants are asked to do a “reality check,” self-distract by carrying out a task or looking elsewhere, or simply block internal affect, activate left IPL/TPJ less often.

The recruitment of IPL in studies of cognitive reappraisal and in the SST study suggests that mechanisms of attentional control based on executive function should be complemented with the contributions that semantic networks may give to emotion regulation (Table 4). This contribution may be accomplished either through their deactivation in proactive control, or through their capacity to support sophisticated semantic encoding of stimuli prior to attentional regulation. This mechanism would be consistent with differences in IPL activity found in borderline personality disorder patients probed with the cognitive reappraisal paradigm (Koenigsberg et al., 2009; Schulze et al., 2011; Lang et al., 2012), who respond to therapeutic approaches that increase the capacity to articulate and semantically encode emotional exchanges (Viviani et al., 2011). It would also be consistent with the observed effect on IPL of psychotherapy, tested with a cognitive reappraisal probe (Goldin et al., 2013).

## Open questions and issues for future studies

This review has not addressed several issues arising from the integration of data on neural substrates of orienting and on emotion regulation. One is the role of the prefrontal parts of the ventral network (Figure 1B). In particular, the VLPFC in the left hemisphere has been associated with voluntary emotion regulation (Ochsner and Gross, 2005), and in the right hemisphere with inhibition of a prepotent response (Aron et al., 2004). Recent studies, however, have cast doubts on the characterization of VLPFC as a locus of control (Sharp et al., 2010). Studies more specifically targeting the relationship between the role of VLPFC in attention and control will be required to shed light on this issue.

A second issue concerns the functional significance of deactivations. In the present review, the hypothesis that deactivations observed in the ventral network were the neural signature of “filters” for incoming stimuli was extended to the emotional domain, and related to the existence of proactive forms of control that constrain processing. However, this hypothesis rests on relatively limited set of data. Clarifying the nature of deactivation may be of particular importance for the clinical neurosciences. A rich PET tradition of early clinical neuroimaging studies has consistently implicated the activation-deactivation balance between ventral and prefrontal dorsal areas in depression (Mayberg et al., 1999) or in processing emotional material (Drevets and Raichle, 1998; Whalen et al., 1998a; Bush et al., 2000; Perlstein et al., 2002). In the medial face of the prefrontal lobes, in particular, dorsal and ventral areas display activation and deactivation during focussed tasks, similar to the dissociation in the parietal lobes. Furthermore, the orbitofrontal cortex hosts representations of affective value of stimuli and expectations of reward or punishment that are used to generate response but are, unlike those in the parietal lobe, largely invariant to sensory features or spatial location (O'Doherty, 2007; Kable and Glimcher, 2009; Elliott et al., 2010).

A third issue concerns the importance of the ventral network for alternative forms of emotion regulation. Investigators have repeatedly noted the existence of forms of emotion regulation that cannot be adequately characterized in terms of a dual process model opposing perceptual salience on the one hand and endogenous attentional control on the other, leading them to formulate the concept of automatic forms of regulation (Bargh and Williams, 2007; Mauss et al., 2007; Phillips et al., 2008). These concepts are broadly consistent with clinical notions of involuntary mental processes handling emotional context, such as the tendency of some patients to avoid psychic painful content. However, difficulties in the operationalization of spontaneous or “automatic” forms of emotion regulation have contributed to hampering progress on its investigation. Here, I have proposed the scrambled sentences task as an empirical model of a form of emotion regulation that takes place spontaneously. However, it is unclear if this task is representative of all forms of emotion regulation that are characterized as automatic in the literature.

## Methods

Because of the considerable diversity of tasks considered in this review, selection of studies based on search words proved of limited utility. These searches were complemented by existing reviews and by systematically checking references of retrieved studies (including subsequent studies citing retrieved studies using online tools such as Google Scholar). Effects in IPL were defined as activations in BA40 and BA39, while the adjacent portion of temporal area BA22 was included as effect in the TPJ. Effects in SPL were defined as activations in BA7.

Studies of attention to emotion (Table 1) are here understood as studies in which cued attention, short term memory or working memory tasks were investigated by varying the existence or absence of emotional tone in stimuli. In short term or working memory tasks emotional variation involved distracters or was incidental to the task. Note that these studies differ from another large category of studies, in which the cognitive load is varied on stimuli that are emotional (these studies provide evidence on the effect of recruitment of cognitive processes on emotional processing, not on the effect of emotional content on the recruitment of attentional processes). Only studies that reported data on healthy participants were considered (including studies on patient populations that reported effects on the healthy separately). For studies presenting an emotional distracter prior to the target stimulus, studies were considered where the interval between distracter and target was less than 500 ms. based on the data by Broadbent and Broadbent (1987) showing no interference at onset asynchronies starting between 480 and 750 ms. Studies with longer asynchronies may be viewed as studies of emotion induction, an issue not considered in the present review. The following studies satisfied these criteria, but were excluded because they omitted to report a whole brain analysis, did not report the emotion vs. neutral contrast, or a combination of both, or were not easily interpretable for the issue at hand: George et al. (1994, 1997), Simpson et al. (2000), Perlstein et al. (2002), Hare et al. (2005), Williams et al. (2005), Etkin et al. (2006), Shafritz et al. (2006), Beneventi et al. (2007), Blair et al. (2007), Mitchell et al. (2007a,b), Dickie and Armony (2008), Lee et al. (2008), Berkman et al. (2009), Siman-Tov et al. (2009), Hart et al. (2010), Padmala and Pessoa (2010), Pereira et al. (2010), Kanske and Kotz (2011b), Sagaspe et al. (2011), Wessa et al. (2013).

Studies on the subliminal presentation of emotional stimuli (Table 2) were selected on the basis of the declared intent of the authors. Methodological studies raise issues on the presentation time that ensures that perception is subliminal (Maxwell and Davidson, 2004). However, only two neuroimaging studies would satisfy stringent criteria for subliminal perception (Liddell et al., 2005; Harmer et al., 2006). Both studies reported no effect in IPL. The following studies were excluded from Table 2 because they omitted the whole brain analysis: Whalen et al. (2004), Pessoa et al. (2006).

In the section on emotion regulation (Table 3), the following studies were excluded from analysis because they omitted the whole brain analysis, did not report on the contrast considered in Table 3, or provided information that could not easily be interpreted in terms of the issues examined here: Schaefer et al. (2002), Ray et al. (2005), Erk et al. (2006), Banks et al. (2007), van Reekum et al. (2007), Abler et al. (2008), Drabant et al. (2009), Ochsner et al. (2009b,a), Kober et al. (2010), Campbell-Sills et al. (2011), Ichikawa et al. (2011), Schulze et al. (2011), Vrtička et al. (2011, 2012, 2013), Lang et al. (2012), Lee et al. (2012), McRae et al. (2012b), Opitz et al. (2012).

Statistical analyses involving logistic regression with repeated measurements were conducted with the function “lmer” (Bates and Maechler, 2009) available within the additional package “lme4” of the freely available software “R” (The R Foundation for Statistical Computing, Vienna, Austria), version 2.14.0. In the model to test localization in the dorsal and ventral parietal lobes (Section Neuroimaging Studies of Voluntary Emotion Regulation), positive or negative finding, as reported in Table 3, was regressed on location (dorsal or ventral) and study (as the grouping variable for repeated measurements). In the test on the tendency of recent studies to report ventral location, report of IPL recruitment was regressed on time of publication of the study. Significance levels reported in the text are two-sided. Models were formulated after inspecting data to quantify effects; they should be therefore understood as explorative.

Figures were prepared with the freely available software Caret (Van Essen et al., 2001; available online from the website http://brainvis.wustl.edu/wiki/index.php/Caret:About).

### Conflict of interest statement

The author declares that the research was conducted in the absence of any commercial or financial relationships that could be construed as a potential conflict of interest.
